# Belongingness in medical students: did it change during lockdown?

**DOI:** 10.1080/10872981.2024.2403807

**Published:** 2024-09-13

**Authors:** Rob Daniels, Eric Buramba, Kato Denis

**Affiliations:** a School of Health and Life Sciences, University of Exeter, Exeter, UK; b Department of Trade Statistics, National Institute of Statistics of Rwanda, Kigali, Rwanda

**Keywords:** Belongingness, legitimate peripheral participation, medical students, United Kingdom, Covid-19, lockdown

## Abstract

Belongingness has been proposed as a potential proxy for legitimate peripheral participation in medical education. Previous studies have shown good internal and external validity for tools designed to measure this variable, with potential use measuring the effectiveness of clinical teaching environments and as a marker of student wellbeing. This study examined changes in belongingness in medical students at the University of Exeter measured in spring 2019 and the equivalent period in 2021, during which COVID-19 related restrictions were in place in the United Kingdom. This study used a validated assessment tool that was self-administered via an online survey platform in 2021. Anonymised data was collected from undergraduate medical students from all years of training and results compared with previous data collected in 2019. The belongingness assessment tool described here had validity in undergraduate medical students studying at the University of Exeter and identified statistically significant changes in belongingness (as measured with this tool) between 2019 and the period during which COVID-19 restrictions were in place. These results suggest that belongingness – in undergraduate medical students fluctuates and varies under different conditions and that there was a statistically significant change during the period of lockdown restrictions. The ability to measure this key facet of educational development has the potential to monitor teaching environments to ensure optimal learning conditions for all students. Further work is required to assess whether the impacts of lockdown restrictions are transient or persist beyond the period of teaching restrictions and to determine any association with academic outcomes.

## Introduction: what is belongingness and how does it contribute to the development of medical students?

Over the course of their university training, medical students undergo a transformation from school-leaver to membership of a profession with highly specialised knowledge, skills and attitudes, combining regulatory aspects and loosely defined professional values. Applying social identity theory as described by Tajfel et al. [1] to this process there are two key phases to developing the professional identity of a doctor. The first stage usually occurs at the start of undergraduate training, when individuals take on the identity of a medical student, a subgroup of the student population who self-identify accordingly. The second stage of transition to the status of qualified doctor represents the end point of a transition over several years, with students expected to demonstrate the professional attitudes expected of the role in the latter stages of their training. This is accentuated by the apprenticeship model of clinical training described by Wenger [2], with students working alongside qualified doctors in a community of practice as they first acquire the identity of a ‘doctor’ and ultimately their final identity within their chosen speciality.

During this period of evolving social-identification students acquire belongingness, defined as the need to be, and perception of being involved with others at differing interpersonal levels … which contributes to one’s sense of connectedness (being part of, feeling accepted, and fitting in), and esteem (being cared about, valued and respected by others), while providing reciprocal acceptance, caring and valuing to others as described by Levett-Jones, Lathlean [3]. The process where students develop this sense of belongingness is therefore likely to be a key component of legitimate peripheral participation, which underpins the structure of functioning clinical teams. The importance of students having a defined role within these teams, both for their education and development of professional identity through increased social capital, has been highlighted in medical students during longitudinal placements [4].

Given this importance, anything that impacts the development of belongingness between individual students and these teams could have lasting effects on the ability of students to acquire the knowledge, skills and values of fully qualified clinicians. Although the ability to measure belongingness in medical students has already been demonstrated [5,6] it is not clear whether belongingness is a trait or state, or whether this varies in response to environmental changes, such as a new curriculum or external factors.

The authors of this paper have previously described a belongingness assessment tool, based on the BES-CPE tool designed by Levett-Jones et al. [3], the Manchester Clinical Placement Index (MCPI) [7], Sense of Belonging Instrument (SOBI-A) [8], and the Dundee Ready Education Environment Measure (DREEM) [9]. This has been shown in medical students in the UK to have internal and external validity and was able to detect statistically significant differences between different clinical teaching environments [5]. This assessment tool generated a numerical score for belongingness rather than a simple binary ‘in/out’ group identity that facilitates study of this area in more depth. The ability to quantify not just academic progress but also the emotional and affective experiences of different students in clinical teaching environments could thus underpin curriculum design but also explore why some students thrive in certain environments and some do not, despite equivalent academic ability.

The COVID-19 pandemic offered an opportunity to investigate whether belongingness varies in response to changes in teaching environments. The second and third periods of national lockdown in the United Kingdom coincided with the first terms at university for the 2020 cohort of medical students, resulting in them having a very different student experience compared to previous cohorts [10]. During that period, all teaching for years 1–4 was online. If belongingness is a core component of both functional communities of practice and formation of social groups, then it is likely that the imposition of lockdown restrictions will impede social group formation and self-identification, and as a result, belongingness, as measured by this tool, would alter in response.

The data collected [5], from all 5 years of the undergraduate medical course at the University of Exeter in 2019, offered the opportunity to repeat the analysis on the equivalent group of students during COVID-19 lockdown in 2021, to test this hypothesis.

## Methods

This study set out to answer the following question:

- Did belongingness (as measured with this tool) in undergraduate medical students differ between 2019 and 2021 in undergraduate medical students studying the same curriculum but under different conditions?

### Study design

Studies of teaching quality and teaching environment are at risk of bias, and one of the researchers in this project is involved in primary care teaching at a design and delivery level. Using a quantitative approach can reduce the impact of any bias in this area, although we do not completely exclude this in the analysis. The researchers were cognisant of this risk during analysis.

The belongingness assessment tool described previously was adapted to remove the lowest ranking items from the previous study [5] to generate 39 items. The tool included three domains: university and peer relationships, most recent secondary care attachment, and most recent primary care attachment. These were included to further explore the findings in the previous study that suggested significant differences between primary care placements and secondary care placements. The survey items are listed in Appendix 1. Given the restrictions on face-to-face meetings during the 2021 study period, data was collected using a self-administered prospective online survey, and the results were then compared with data collected using the same survey instrument in 2019 in the equivalent student group.

### Setting

The structure of the undergraduate medical program has an initial phase of core medical sciences with some clinical exposure, followed by a second predominantly clinical phase. This study used data previously collected in 2019 by the authors (5) using the belongingness assessment tool in Appendix 1, with students attending whole cohort teaching sessions invited to participate. Medical students in years 1–5 at the University were invited to participate between January and April 2019 and the same period in 2021 collected through an online portal due to teaching restrictions in place at that time.

### Participants

All undergraduate medical students registered at the University of Exeter at the time of the study were given the opportunity to participate. There were no financial rewards or penalties for not participating, and responses were anonymised.

### Recruitment

All medical students were eligible to participate. The 2021 survey was publicised by student peer ambassadors and through social media, given that there were few formal in-person teaching activities. The 2019 survey was distributed at in-person cohort teaching events.

### Data collection and analysis

Data for the 2021 cohort was collected using the online portal Online Surveys [11] between 26 February 2021 and 17 April 2021. The 2019 data were collected at in-person teaching events between March and April 2019. Data was analysed using Stata [12] and Jamovi [13].

## Results

Characteristics of respondents are shown below in Table 1.

**Table 1.** Characteristics of respondents.

	2019	2021
Response rate	31%	19%
Gender	Sample (%)	Sample (%)
Female	85(55.2)	114(70.4)
Male	62(40.3)	42(25.9)
Non-binary	-	1(0.6)
Not given	7(4.5)	5(3.1)
Ethnicity	Sample (%)	Sample (%)
Caucasian	113(73.4)	128(79.0)
Non-Caucasian	33(21.4)	30(18.5)
Not given	8(5.2)	4(2.5)
Migration Background		
Year	Sample (%)	Sample (%)
Year 1	34(22.1)	32(19.8)
Year 2	20(13.0)	47(29.0)
Year 3	36(23.4)	23(14.2)
Year 4	23(14.9)	27(16.7)
Year 5	41(26.6)	29(17.9)
Not given	-	4(2.5)
English as First language	Sample (%)	Sample (%)
Yes	130(84.4)	137(84.6)
No	16(10.4)	21(13.0)

### Validation of belongingness assessment tool in UK medical students

In total 154 students in 2019 and 162 students completed the survey in 2021. Students were recruited by year group student ambassadors in the UK through online forums and cohort teaching events. The belongingness tool comprising 39 questions showed a satisfactory overall internal consistency in both cohorts and exploratory factor analysis of the data set was carried out. For the 2019 cohort, Kaiser-Meyer-Olkin was 0.869 and for the 2021 cohort 0.862, indicating adequate sampling, and Cronbach’s alpha was 0.940 for 2019 cohort, and 0.931 for 2021.

### Did belongingness (as measured by this tool) in undergraduate medical students alter between 2019 and 2021 in undergraduate medical students in the UK studying the same curriculum but under different conditions?

**Figure 1.**
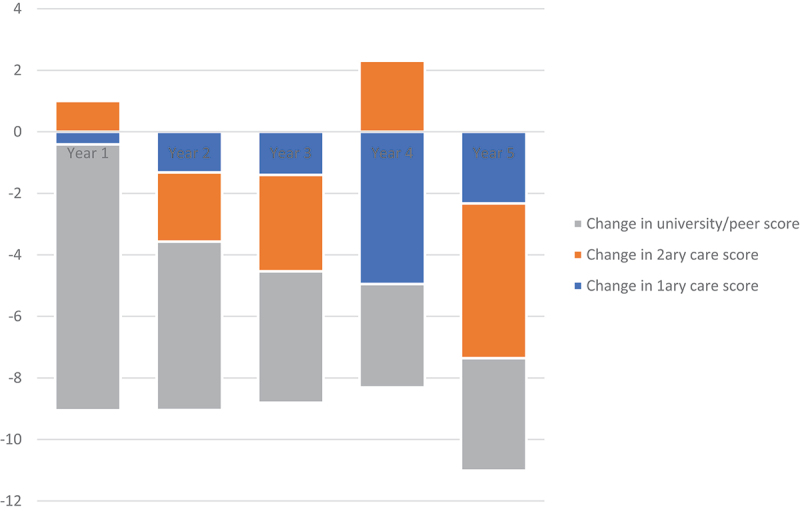
Change in each component of belongingness, for each year group between 2019 and 2021.

Full results are shown in Tables 2–4. Statistically significant differences were seen in all components of belongingness, as measured by this tool between 2019 and 2021. The largest decrease was seen in peer/university score (9.4%, *p* = 0.0000) and the smallest increase was in secondary care score (3.2%, *p* = 0.0501). This may relate to the changes in teaching methods within each care sector, as a result of the COVID-19 lockdown. These results are shown in Table 2 and Figure 1, below.

**Table 2.** Changes in belongingness for each subset of belongingness score, 2019 vs 2021.

	Year 1: % change p value)	Year 2: % change (p value)	Year 3: % change (p value)	Year 4: % change (p value)	Year 5: % change (p value)	Aggregate score (all years): % change (p value)
Change in University/peer score, 2019 vs 2021	−17.9% (0.0001)	−4.0% (0.2434)	−8.7% (0.0959)	−6.8% (0.1237)	−7.2% (0.1233)	−9.4% (0.0000)
Change in secondary care score, 2019 vs 2021	+2.1% (0.775)	−4.7% (0.3951)	−6.9% (0.0975)	+5.4% (0.3912)	−9.8% (0.0063)	−3.2% (0.0501)
Change in primary care score, 2019 vs 2021	−0.8% (0.7972)	−2.6% (0.6588)	−2.6% (0.5883)	−9.1% (0.1284)	−3.9% (0.0990)	−5.3% (0.0077)
Change in total score, 2019 vs 2021	−5.3% (0.0496)	−3.7% (0.3477)	−6.0% (0.1110)	−4.1% (0.2929)	−6.8% (0.0057)	−6.0% (0.0000)

**Table 3.** Changes in belongingness for different demographic groups.

	Difference (%) in male students (p value)	Difference (%) in female students (p value)	Difference (%) in caucasian students (p value)	Difference (%) in non-caucasian students (p value)	Difference (%) in anglophone students (p value)	Difference (%) in non-anglophone students (p value)
Change in University/peer score 2019 vs 2021	−12.7% (0.0002)	−9.4% (0.0001)	−9.7% (0.0000)	−14.3% (0.0007)	−9.3% (0.0000)	−12.4% (0.0170)
Change in secondary care score, 2019 vs 2021	−6.9% (0.0435)	−2.8% (0.2140)	−3.8% (0.081)	−5.9% (0.2254)	−3.8% (0.0752)	−1.4% (0.8039)
Change in primary care score, 2019 vs 2021	−6.8% (0.0474)	−6.0% (0.0211)	−5.5% (0.0087)	−8.0% (0.0414)	−5.4% (0.0076)	−5.4% (0.2888)
Change in total score, 2019 vs 2021	−8.8% (0.0020)	−6.2% (0.0012)	−6.4% (0.0000)	−9.3% (0.0085)	−6.2% (0.0001)	−6.4% (0.0751)

**Table 4.** Longitudinal change in belongingness scores for matched 2019 year groups vs same group of students in 2021.

	Year 1 (2019) vs Year 3 (2021): % change (p value)	Year 2 (2019) vs Year 4 (2021): % change (p value)	Year 3 (2019) vs Year 5 (2021): change (p value)
Change in University/peer score	−11.8% (p = 0.007)	−2.9% (p = 0.287)	−2.3% (p = 0.294)
Change in secondary care score	−12.4% (p = 0.008)	−7.0% (p = 0.082)	+3.8% (p = 0.214)
Change in primary care score	−0.2% (p = 0.485)	−2.5% (p = 0.324)	+8.7% (p = 0.018)
Change in total score	−7.9% (p = 0.038)	−4.2% (p = 0.166)	+3.5% (p = 0.137)

Comparing equivalent year groups in 2019 and 2021, differential effects were seen for different year groups within the student population. For students in year 1, peer/university score fell by 17.9% (*p* = 0.0001)and total score by 5.3% (0.0496). The decline in the peer/university score accounted for 93.5% of the reduction in total score for year 1. The decline in peer/university score was less marked for other years and did not reach statistical significance.

Differences were also seen for students in year 5, mainly in secondary care score and total score, with a 9.8% (*p* = 0.0063) decrease in secondary care score. Scores for total belongingness in both 2019 and 2021 were significantly higher in year 5 than in other years.

Statistically significant changes in primary care, peer/university and total score were seen for both non-Caucasian and Caucasian students, and for male and female students. For students who spoke English as a second language, the only significant difference was in Peer/university score. This may represent the small number of students who spoke English as a second language (10.4% in 2019 and 13.0% in 2021) affecting statistical significance.

Three of the year groups were represented in both surveys, with the year 1 cohort in 2019 being in their 3^rd^ year in 2021, year 2 in their 4^th^ year and year 3 in their fifth year in 2021. This allowed the scores for these groups to be tracked between the two study periods. Statistically significant changes were observed for the following groups:

- Year 1 (2019)-Year 3 (2021): Total belongingness decreased by 7.9% (*p* = 0.038), peer university score decreased by 11.8% (*p* = 0.007) and secondary care score decreased by 12.4% (*p* = 0.008)
- Year 3 (2019)-Year 5(2021): Primary care score increased by 8.7% (*p* = 0.018).

## Discussion

This data presented here confirm that the belongingness assessment tool in Appendix 1:

- Identified statistically significant differences in belongingness (as measured with this tool) in undergraduate medical students during a period of significant change to student teaching conditions and student lifestyle
- Supports the hypothesis that a key phase of social identity formation is likely to occur at the outset of undergraduate medical training.

The significant decline in peer/university belongingness seen in year 1 compared to later years of study may reflect the loss of the initial phase of medical student identity formation, when students come together for the first time, forming supportive academic and social peer groups. The impact of COVID-19 related restrictions on opportunities to socialise within timetabled teaching sessions and in informal settings, is likely to have had a detrimental effect on the formation of these networks compared to previous years. Students in later years will have had the benefit of this period of socialisation in earlier years which may have a lasting protective effect on belongingness.

When year groups were analysed longitudinally (i.e., the first-year students in 2019 became the third year students in 2021), significant differences in peer/university scores were seen only in students in year 1 in 2019. This suggests that as belongingness develops, it may provide a degree of resilience, further highlighting the importance of this concept as a consideration in clinical teaching, as described by Vivekananda-Schmidt and Sandars [6] and Gopalan et al. [14]. Further work to quantify this facet of belongingness going forward in this cohort may shed more light on the potential medium to long-term impacts of the change in belongingness noted in year 1 students.

Differences seen for students in year 5 may reflect the fact that students in their penultimate term as medical students in Exeter are effectively working in the final stage of their apprenticeship, having completed the majority of summative assessments, and traditionally working alongside junior doctors doing many of the same tasks and holding a similar status as newly qualified doctors. COVID-19 related restrictions may have prevented these tasks being carried out to the same degree as in previous years and this impacted the extent to which the 2021 cohort felt themselves to be legitimate members of their clinical teams. The data described in this study for total belongingness in both 2019 and 2021 are significantly higher in year 5 compared to other year groups, which is similar to longitudinal studies of measures of belongingness in American medical students [15]. These findings support the concept of belongingness peaking as students approach the end of their training, at which point they feel validated as members of the medical profession, by virtue of their formal qualification.

These findings support the potential for monitoring the teaching experience when there is an internal or external change, such as a new curriculum. This will help ensure the optimal environment for medical students to develop within communities of practice, with belongingness as a proxy for legitimate peripheral participation.

## Strengths and weaknesses and areas for further study

Further work to explore the relationship between belongingness and academic performance in individual students would be useful to determine the extent to which these factors are related. It would also be useful to collect data on the scores for individual speciality placements, to determine the impact of a short attachment in an individual environment on belongingness, and the usefulness of belongingness scores as tools to monitor teaching placements. This study benefitted from the ability to analyse a similar population of students at the same stage of the academic year, with the main difference being COVID-19 related changes in teaching environment. This reduced but did not eliminate potential confounding influences. This study also looks at belongingness in three facets of medical education: primary care placement, secondary care placement and university experience. The relationship between individual personality characteristics, belongingness and specialism preference has not been addressed here. It is likely that students will have a natural inclination to a certain discipline based on interests and skillset, and the extent to which this is thwarted or reinforced by perceptions of in/out group status for individual students merits further qualitative investigation.

One of the weaknesses of this study is the relatively low number of students in ethnic minority and first-generation groups. Evidence from elsewhere [15] suggests that these groups may be disproportionately affected by changes in belongingness. Further study in student groups with a higher proportion of patients in these groups will shed more light on this. The ability of this tool to identify differences between different student groups suggests there may be some merit in using this to identify struggling postgraduate trainees, such as newly arrived international medical graduates who now make up 34% of UK GP trainees [16].

The importance of belongingness and perceived ‘fit’ has been shown to have a positive relationship with retention, well-being and speciality selection [17], and this is particularly evident in specialities that are the least diverse, such as orthopaedics [18]. The role of medical school experience as an opportunity to influence student feelings of belongingness both within individual specialities and in the wider medical school is likely to be an important determinant of future engagement or attrition. The significant changes noted in belongingness, particularly in peer/university score, between 2019 and 2021 therefore need further investigation to determine whether this was a transient reaction to circumstances at the time of the study or whether the significant decline noted in year 1 persists with long-term impacts on this aspect of medical education.

## Conclusion

The belongingness assessment tool described in this paper and elsewhere has good internal validity and identified differences in belongingness between different demographic groups and over time, suggesting it may be a useful tool to quantify student learning experience in undergraduate medical training environments. Statistically significant differences were seen between different groups during COVID-19 related restrictions and it is likely that these reflect the impact of these restrictions on the ability of medical students to learn effectively. In particular, the potential impact on the cohort who started their medical education in 2020 warrants further study to determine if these changes are short term or persist into later years with the potential to impede effective learning. Awareness of the disproportionate impact of COVID-19 restrictions on different student groups will allow appropriate remedial action to be considered, if these changes prove to be persistent.

## Supplementary Material

Supplemental Material

## Data Availability

The datasets used and/or analysed during the current study are available from the corresponding author on reasonable request.
