# Supplementary material for: Belongingness in medical students: did it change during lockdown?
Source: Med Educ Online. 2024 Sep 13;29(1):2403807. doi: 10.1080/10872981.2024.2403807 (PMC11404386; doi:10.1080/10872981.2024.2403807)
Supplement: Supplemental Material [file ZMEO_A_2403807_SM7801.docx]

**Appendix 1: questionnaire items**

**Undergraduate Medical Student Belongingness Questionnaire**

This questionnaire is designed to assess how strongly undergraduate medical students feel a sense of belongingness during clinical placements, and what factors affect this. A better understanding of this will help understand the student experience and improve the quality of clinical placement experience.

Belongingness is defined as the extent to which an individual feels:

(a) secure, accepted, included, valued and respected by a defined group,

(b) connected with or integral to the group, and

(c) that their professional and/or personal values are in harmony with those of the group.

(Levett-Jones and Lathlean, 2008)

This can apply to a student’s relationship with their student cohort, their medical school and their relationship to the wider profession.

**Instructions**

For each section of the questionnaire, read each statement and then select the response that best indicates how often the statement is true for you.

For example, if you eat dessert after dinner almost every night you would select ‘Often True’. If you rarely eat dessert you would select ‘Rarely True’.

For each statement:

• Please answer every statement, even if one seems similar to another one

• Answer each statement quickly.

• Think generally about your most recent clinical placement experiences when considering your responses to the statements.

In the statements below, ‘placement/s’ refers to your clinical attachments in hospital wards or GP practices.

**Section 1: Demographic information**

This data is optional, but will allow us to better understand differences between groups of students. All responses will be anonymised.

Year of study:

Ethnic group: White British/Asian/Afro-Caribbean/Other/Rather not say

Gender: M/F/Rather not say

Is English your first language? Yes/No

**Section 2: Student feelings of belongingness**

| **A** | **Relationship with the University, Medical School and medical profession** | **These questions relate to your current feelings of belongingness towards the university, medical school and wider medical profession.** |
| --- | --- | --- |
| 01 | I feel that there are other students at the medical school like me | Never True    Rarely True    Sometimes True    Often True    Always True |
| 02 | I feel that there are other students at the university like me | Never True    Rarely True    Sometimes True    Often True    Always True |
| 03 | I feel a sense of belongingness to the medical school | Never True    Rarely True    Sometimes True    Often True    Always True |
| 04 | I feel a sense of belongingness to the university | Never True    Rarely True    Sometimes True    Often True    Always True |
| **B** | **Working relationships with other medical students on clinical placements** | **These questions relate to your relationship with your fellow students on clinical placement in the current academic year** |
| 05 | When I walk up to a group of fellow students on a clinical placement I feel welcomed | Never True    Rarely True    Sometimes True    Often True    Always True |
| 06 | I feel understood by my fellow students (either in professional or personal sense) | Never True    Rarely True    Sometimes True    Often True    Always True |
| 07 | I feel that my fellow students are interested in my life outside my studies | Never True    Rarely True    Sometimes True    Often True    Always True |
| 08 | I feel that I could approach my fellow students outside placements | Never True    Rarely True    Sometimes True    Often True    Always True |
| 09 | I respect the other students I work with on placements | Never True    Rarely True    Sometimes True    Often True    Always True |
| 10 | I think that the students I work with on placements respect me | Never True    Rarely True    Sometimes True    Often True    Always True |
| 11 | Other students on my placements invite me to eat lunch/dinner with them | Never True    Rarely True    Sometimes True    Often True    Always True |
| 12* | I am uncomfortable attending social functions involving fellow students on placements because I feel like I don’t belong | Never True    Rarely True    Sometimes True    Often True    Always True |
| 13 | I feel confident in my knowledge and ability compared to my fellow students | Never True    Rarely True    Sometimes True    Often True    Always True |

| **C** | **Placement working environment for your most recent secondary care placement .** | **These questions relate to your feelings of belongingness on your most recent placement in secondary care (i.e. Hospital based)** |  |
| --- | --- | --- | --- |
|  | **Speciality: (leave blank if you would rather not say)** |  |  |
| (i) | *Relationship with senior clinical staff (doctors not in training, e.g. consultants)* |  |  |
| 14 | I felt that the senior clinical staff treated me as an equal | Never True    Rarely True    Sometimes True    Often True    Always True |  |
| 15 | I would have felt comfortable asking for support or advice from senior clinical staff when I needed it | Never True    Rarely True    Sometimes True    Often True    Always True |  |
| 16 | I felt able to actively participate in clinical teaching e.g. by asking questions | Never True    Rarely True    Sometimes True    Often True    Always True |  |
| 17 | I felt the senior clinical staff treated me as an individual | Never True    Rarely True    Sometimes True    Often True    Always True |  |
| 18 | I felt the senior clinical staff knew who I was | Never True    Rarely True    Sometimes True    Often True    Always True |  |
| (ii) | *Relationship with other staff (doctors in training, nurses, therapists, admin staff)* |  |  |
| 19 | When I walked up to the staff on the first day of this placement, I felt welcomed | Never True    Rarely True    Sometimes True    Often True    Always True |  |
| 20 | I felt that I had a role in the wider clinical team (non-medical members e.g. nurses, admin staff) | Never True    Rarely True    Sometimes True    Often True    Always True | |
| 21 | I felt respected as a medical student by the wider clinical team (non-medical members e.g. nurses, admin staff) | Never True    Rarely True    Sometimes True    Often True    Always True | |
| 22* | The clinical staff (doctors) on the placement made me feel like I was wasting their time | Never True    Rarely True    Sometimes True    Often True    Always True | |
| 23 | I felt that the clinical staff were happy to make time to teach me practical procedures | Never True    Rarely True    Sometimes True    Often True    Always True | |
| 24* | I was uncomfortable attending meetings e.g. ward rounds on the placement because I felt that I didn’t belong | Never True    Rarely True    Sometimes True    Often True    Always True | |
| 25* | I felt discriminated against on placement (you can provide more details of this at the end | Never True    Rarely True    Sometimes True    Often True    Always True | |
| 26 | I felt a sense of belongingness to the team on this clinical placement | Never True    Rarely True    Sometimes True    Often True    Always True | |

| **D** | **Perception of your most recent clinical placement in primary care** | **These questions relate to your feelings of belongingness on your most recent placement in primary care (ie.. General Practice)** |
| --- | --- | --- |
| (i) | *Relationship with senior clinical staff (Qualified GP’s)* |  |
| 27 | I felt that the senior clinical staff treated me as an equal | Never True    Rarely True    Sometimes True    Often True    Always True |
| 28 | I would have felt comfortable asking for support or advice from senior clinical staff when I needed it | Never True    Rarely True    Sometimes True    Often True    Always True |
| 29 | I felt able to actively participate in clinical teaching e.g. by asking questions | Never True    Rarely True    Sometimes True    Often True    Always True |
| 30 | I felt the senior clinical staff treated me as an individual | Never True    Rarely True    Sometimes True    Often True    Always True |
| 31 | I felt the senior clinical staff knew who I was | Never True    Rarely True    Sometimes True    Often True    Always True |
| (ii) | *Relationship with other staff (doctors in training, nurses, receptionists, admin staff)* |  |
| 32 | When I walked up to the staff on the first day of this placement, I felt welcomed | Never True    Rarely True    Sometimes True    Often True    Always True |
| 33 | I felt that I had a role in the wider clinical team (non-medical members e.g. nurses, admin staff) | Never True    Rarely True    Sometimes True    Often True    Always True |
| 34 | I felt respected as a medical student by the wider clinical team (non-medical members e.g. nurses, admin staff) | Never True    Rarely True    Sometimes True    Often True    Always True |
| 35* | The clinical staff (doctors) on the placement made me feel like I was wasting their time | Never True    Rarely True    Sometimes True    Often True    Always True |
| 36 | I felt that the clinical staff (doctors) were happy to make time to teach me practical procedures | Never True    Rarely True    Sometimes True    Often True    Always True |
| 47* | I was uncomfortable attending meetings e.g. practice meetings on placements because I felt like I don’t belong | Never True    Rarely True    Sometimes True    Often True    Always True |
| 38* | I felt discriminated against on placement (you can provide more details of this at the end) | Never True    Rarely True    Sometimes True    Often True    Always True |
| 39 | I felt a sense of belongingness to the team on this clinical placement | Never True    Rarely True    Sometimes True    Often True    Always True |

Any other comments:
